# Artificial Intelligence in Febrile Neutropenia: From Risk Scores to Real-Time Clinical Decision Support

**DOI:** 10.7759/cureus.112346

**Published:** 2026-07-09

**Authors:** Kunj R Ghantiwala, Ruchik Kevadiya, Pradeep Pentapurthy, Michael V Kolar, Vesna Tegeltija

**Affiliations:** 1 Medicine, Gujarat Medical Education and Research Society (G.M.E.R.S.) Medical College, Navsari, IND; 2 Medicine, Government Medical College, Bhavnagar, IND; 3 Internal Medicine, Henry Ford Health System, Rochester Hills, USA; 4 Internal Medicine, Wayne State University/Henry Ford Rochester Hospital, Rochester Hills, USA; 5 Medicine, Saint Martinus University Faculty of Medicine, Willemstad, CUW; 6 Internal Medicine, Wayne State University School of Medicine, Rochester Hills, USA

**Keywords:** artificial intelligence, clinical decision support systems, febrile neutropenia, machine learning, oncology

## Abstract

Febrile neutropenia remains one of the most urgent and clinically challenging complications in oncology care, where timely recognition and decision-making can significantly influence outcomes. While traditional risk assessment tools have long supported clinicians in stratifying patients, they are limited in their ability to adapt to the complexity and rapidly changing nature of clinical situations. In this context, artificial intelligence (AI) is increasingly being explored as a way to complement existing approaches and support more responsive and individualized care.

This editorial discusses how the role of AI in febrile neutropenia is evolving, moving from static risk scoring systems toward the possibility of real-time clinical decision support. It highlights both the potential of these tools to enhance clinical workflows and the practical challenges that still need to be addressed before widespread adoption. As these technologies continue to develop, careful integration into clinical practice may help improve how clinicians assess risk and manage patients with febrile neutropenia.

## Editorial

Febrile neutropenia (FN) remains one of the most consequential complications encountered in oncology practice; it frequently requires urgent clinical evaluation and prompt therapeutic management. Despite advances in cancer therapy and supportive care, FN continues to pose substantial challenges because of its unpredictable clinical course and potential for rapid progression to severe infection and other life-threatening complications. Clinicians are often required to make critical decisions regarding hospitalization, empirical antimicrobial therapy, and monitoring strategies within a short time frame, underscoring the importance of accurate risk assessment and timely intervention [1].

For decades, clinical prediction models such as the Multinational Association for Supportive Care in Cancer (MASCC) risk index and the Clinical Index of Stable FN (CISNE) have provided a structured framework for risk stratification in FN [2]. While these tools have contributed significantly to standardizing patient assessment and management, they remain limited by their reliance on predefined variables and static scoring systems. Existing prediction models continue to face challenges related to performance, validation, and applicability across diverse patient populations and healthcare settings, as highlighted by a recent systematic review [2]. The complexity of FN reflects a dynamic interaction among host factors, disease characteristics, treatment exposures, and evolving infectious risks. At the same time, traditional approaches may not fully capture the realities of clinical decision-making in modern oncology practice [2].

The growing availability of electronic health records and large clinical datasets has fueled increasing interest in artificial intelligence (AI) and machine learning (ML) as potential solutions to these limitations [1]. Unlike conventional risk assessment tools, AI-based models can process large volumes of heterogeneous clinical data, identify complex relationships among variables, and generate individualized predictions that evolve as new information becomes available [1]. This capability is particularly attractive in FN, where management decisions are often time-sensitive and depend on multiple interacting clinical factors [1].

Emerging evidence suggests that AI may have meaningful applications across several aspects of FN management. ML models have demonstrated promising performance in predicting bloodstream infections among patients with hematologic malignancies presenting with FN, potentially enabling earlier identification of individuals at increased risk for infectious complications [3]. Earlier recognition of high-risk patients could facilitate more targeted monitoring strategies and timely therapeutic interventions, thereby improving clinical efficiency and resource utilization [3].

Beyond infection prediction, AI is increasingly being explored as a tool to support therapeutic decision-making. Recent investigations have evaluated ML algorithms designed to assist with empirical antibiotic selection by incorporating patient-specific characteristics, previous colonization status, and antimicrobial resistance patterns [4]. Similarly, AI-driven antimicrobial guidance systems have demonstrated the feasibility of supporting empirical treatment decisions in patients with hematologic malignancies and FN [5]. These developments illustrate an important evolution in the role of AI from simply predicting risk toward actively informing clinical management [4,5].

Despite these encouraging advances, important challenges remain before AI can be routinely incorporated into FN care. Many existing models have been developed using retrospective datasets, which introduces inherent risks of selection bias, missing data bias, and confounding. In addition, several models are derived from single-center or highly selected patient populations, limiting their generalizability to broader, real-world clinical settings [1]. Variations in patient demographics, disease severity, institutional practices, and microbiological epidemiology may significantly influence model performance when applied outside their original development environments. These factors raise important concerns regarding external validity and highlight the need for rigorous multicenter validation before clinical implementation [1].

Beyond methodological limitations, issues related to interpretability and transparency remain major barriers to clinical adoption. Many ML models function as “black-box” systems, offering limited insight into how predictions are generated [1]. This lack of explainability can reduce clinician trust, particularly in high-stakes clinical scenarios such as FN where treatment decisions must be made rapidly. As a result, even highly accurate algorithms may face resistance if their decision-making processes cannot be clearly understood or clinically justified [1].

Another critical consideration is whether improved predictive performance translates into meaningful improvements in patient outcomes. The success of AI in healthcare should not be measured solely by statistical accuracy but by its ability to meaningfully enhance clinical decision-making, improve timeliness of intervention, and optimize patient outcomes. Practical implementation challenges, including integration into electronic health record systems, workflow disruption, and clinician acceptance, will ultimately determine whether these technologies achieve real-world impact. Without careful deployment strategies, even highly sophisticated models risk remaining confined to research environments [1].

Conclusion

AI represents a promising frontier in the management of FN. Emerging applications in infection prediction, risk stratification, and antimicrobial decision support have highlighted the potential of AI to address important limitations of traditional approaches. However, substantial challenges related to validation, interpretability, and implementation remain. As the field continues to evolve, careful evaluation and thoughtful integration of AI into clinical practice will be necessary to ensure that these technologies improve patient outcomes while preserving the central role of clinician judgment in supportive oncology care.

## References

[1] Gallardo-Pizarro A, Peyrony O, Chumbita M (2024). Improving management of febrile neutropenia in oncology patients: the role of artificial intelligence and machine learning. Expert Rev Anti Infect Ther.

[2] Sheehy J, Gallanagh M, Sullivan C, Lane S (2025). Clinical prediction models for febrile neutropenia and its outcomes: a systematic review. Support Care Cancer.

[3] Gallardo-Pizarro A, Teijón-Lumbreras C, Monzo-Gallo P (2025). Development and validation of a machine learning model for the prediction of bloodstream infections in patients with hematological malignancies and febrile neutropenia. Antibiotics.

[4] Gallardo-Pizarro A, Teijón-Lumbreras C, Aiello TF (2026). Towards personalised empirical antibiotic therapy in febrile neutropenia: a theoretical model based on machine learning and prior colonisation with multidrug-resistant gram-negative bacilli - a retrospective proof-of-concept cohort study. Ther Adv Infect Dis.

[5] Hoashi K, Matsumoto K, Kiyasu J (2025). Machine learning model to guide empirical antimicrobial therapy in febrile neutropenic patients with hematologic malignancies. Anticancer Res.

